# Clinical improvement of DM1 patients reflected by reversal of disease-induced gene expression in blood

**DOI:** 10.1186/s12916-022-02591-y

**Published:** 2022-11-10

**Authors:** Remco T. P. van Cruchten, Daniël van As, Jeffrey C. Glennon, Baziel G. M. van Engelen, Peter A. C. ‘t Hoen, the OPTIMISTIC consortium, the ReCognitION consortium, K Okkersen, C Jimenez-Moreno, S Wenninger, F Daidj, S Cumming, R Littleford, D G Monckton, H Lochmüller, M Catt, C G Faber, A Hapca, P T Donnan, G Gorman, G Bassez, B Schoser, H Knoop, S Treweek, Derick G. Wansink, Francis Impens, Ralf Gabriels, Tine Claeys, Aymeric Ravel-Chapuis, Bernard J. Jasmin, Niamh Mahon, Sylvia Nieuwenhuis, Lennart Martens, Petr Novak, Denis Furling, Arie Baak, Genevieve Gourdon, Alex MacKenzie, Cecile Martinat, Nafisa Neault, Andreas Roos, Elise Duchesne, Renee Salz, Rachel Thompson, Sandrine Baghdoyan, Anu Mary Varghese, Paul Blom, Sally Spendiff, Alexander Manta

**Affiliations:** 1 Center for Molecular and Biomolecular Informatics, Radboud Institute for Molecular Life Sciences, Radboud University Medical Center, Nijmegen, The Netherlands; 2 Department of Neurology, Donders Institute for Brain, Cognition and Behaviour, Radboud University Medical Center, Nijmegen, The Netherlands; 3 Conway Institute of Biomolecular and Biomedical Research, School of Medicine, University College Dublin, Dublin, Ireland

**Keywords:** Myotonic dystrophy type 1, Biomarker, RNA-seq, Peripheral blood, Therapeutic Response, Lifestyle intervention

## Abstract

**Background:**

Myotonic dystrophy type 1 (DM1) is an incurable multisystem disease caused by a CTG-repeat expansion in the DM1 protein kinase (*DMPK*) gene. The OPTIMISTIC clinical trial demonstrated positive and heterogenous effects of cognitive behavioral therapy (CBT) on the capacity for activity and social participations in DM1 patients. Through a process of reverse engineering, this study aims to identify druggable molecular biomarkers associated with the clinical improvement in the OPTIMISTIC cohort.

**Methods:**

Based on full blood samples collected during OPTIMISTIC, we performed paired mRNA sequencing for 27 patients before and after the CBT intervention. Linear mixed effect models were used to identify biomarkers associated with the disease-causing CTG expansion and the mean clinical improvement across all clinical outcome measures.

**Results:**

We identified 608 genes for which their expression was significantly associated with the CTG-repeat expansion, as well as 1176 genes significantly associated with the average clinical response towards the intervention. Remarkably, all 97 genes associated with both returned to more normal levels in patients who benefited the most from CBT. This main finding has been replicated based on an external dataset of mRNA data of DM1 patients and controls, singling these genes out as candidate biomarkers for therapy response. Among these candidate genes were *DNAJB12*, *HDAC5*, and *TRIM8*, each belonging to a protein family that is being studied in the context of neurological disorders or muscular dystrophies. Across the different gene sets, gene pathway enrichment analysis revealed disease-relevant impaired signaling in, among others, insulin-, metabolism-, and immune-related pathways. Furthermore, evidence for shared dysregulations with another neuromuscular disease, Duchenne muscular dystrophy, was found, suggesting a partial overlap in blood-based gene dysregulation.

**Conclusions:**

DM1-relevant disease signatures can be identified on a molecular level in peripheral blood, opening new avenues for drug discovery and therapy efficacy assessments.

**Supplementary Information:**

The online version contains supplementary material available at 10.1186/s12916-022-02591-y.

## Background

Myotonic dystrophy type 1 (DM1) is a neuromuscular disease with a worldwide average prevalence of around 1 in 8000 people and a high unmet clinical need [1]. DM1 is considered the most frequently occurring adult-onset form of muscular dystrophy. This degenerative multisystem disease is characterized by a wide range of symptoms including myotonia, muscle weakness and dystrophy, fatigue, apathy, cataracts, obesity, and insulin resistance. Next to a severe decrease of life quality, DM1 patients suffer from a reduced life expectancy mostly due to problems with cardiac and respiratory function. Currently, no curative therapy exists.

DM1 is caused by the expansion of a CTG trinucleotide microsatellite repeat in the 3′ UTR of the DM1 protein kinase (*DMPK*) gene [2–4]. Unaffected individuals carry up to approximately 37 CTG triplets in *DMPK*, while in DM1 patients this ranges from 50 to even a few thousand repetitions. Depending on the inherited repeat length, DM1 can become manifest at birth or early in life but more frequently becomes apparent in adulthood [1]. In general, the disease manifestation is earlier and more severe with longer repeat expansions. Interruption of the CTG repeat by variants such as CCG or CGG is associated with milder symptoms [5]. The expanded CTG repeat is thought to cause disease mainly via an mRNA gain-of-function mechanism, in which aberrant hairpin structures formed by long CUG repeats are central [6–9]. Directly or indirectly, these hairpin structures dysregulate the function of RNA binding proteins from the muscleblind-like (MBNL) and CUGBP Elav-like (CELF) families, leading to widespread disturbed RNA processing and consequently altered functions of various proteins [10–12]. Although proven to be the disease-causing mutation, clinical symptoms of DM1 are only moderately associated with the CTG repeat or the dysregulation of specific proteins which suggests an involvement of other mechanisms in symptom expression [13–16].

While there are many promising therapeutic oligonucleotides, small molecule drugs, and gene therapies in the (pre) clinical pipeline for some of the signs and symptoms of DM1, none is expected to reach widespread clinical application soon. Physical training and increasing activity are currently being applied to relieve DM1 symptoms with marked improvements in relatively mildly affected DM1 patients [17, 18], which has furthermore shown to induce biochemical responses in DM1 mouse models [19, 20].

The to-date largest clinical trial in DM1 was OPTIMISTIC: Observational Prolonged Trial In Myotonic dystrophy type 1 to Improve Quality of Life-Standards, a Target Identification Collaboration [18]. The OPTIMISTIC clinical trial included over 250 well-characterized DM1 patients from four centers in Europe, where the effects of cognitive behavioral therapy (CBT) and optional graded exercise therapy were closely monitored over 16 months via more than twenty outcome measures. Notably, the CBT intervention was tailored towards the specific needs of the patient in a shared decision-making process between the patient and the psychotherapist, allowing for a personalized intervention. The trial has shown significant, yet heterogenous improvements for various signs and symptoms, as well as the capacity for social activity and participation in DM1 [21].

Here, we set out to find molecular profiles associated with the disease-causing CTG repeat and therapy response based on full blood mRNA sequencing before and after the CBT intervention of 27 patients from the OPTIMISTIC cohort. Given the accessibility of peripheral blood, it has increasingly been used for the successful identification of disease biomarkers for a variety of neurological and psychiatric disorders such as Duchenne muscular dystrophy (DMD), Huntington’s disease, major depressive disorder, and DM1 [22–25]. Furthermore, the multisystem nature of DM1 is known to be reflected by various laboratory abnormalities of blood samples, supporting the relevance of peripheral blood for the identification of disease-relevant information [26]. We analyzed gene expression levels as a function of CTG repeat size (as a proxy for disease load/severity) and of the therapy response. Next, we combined these findings and compared the results to various previously published datasets. We were able to identify 608 genes significantly associated with the CTG repeat and further illustrate that 97 of these genes returned towards more normal expression levels in clinical CBT responders.

## Methods

### The cognitive behavioral therapy intervention

Patients of the OPTIMISTIC intervention arm were treated with a personalized form of CBT. The customization of the intervention was based on a selection of different treatment modules: regulating sleep-wake patterns, compensating for the reduced patient initiative, formulating helpful beliefs about fatigue and myotonic dystrophy type 1, optimizing social interactions, and coping with pain [18]. The individual module selection was made based on a shared decision-making process between experienced and specifically trained CBT therapists and patients.

### Sample source and patient sampling

**Fig. 1:**
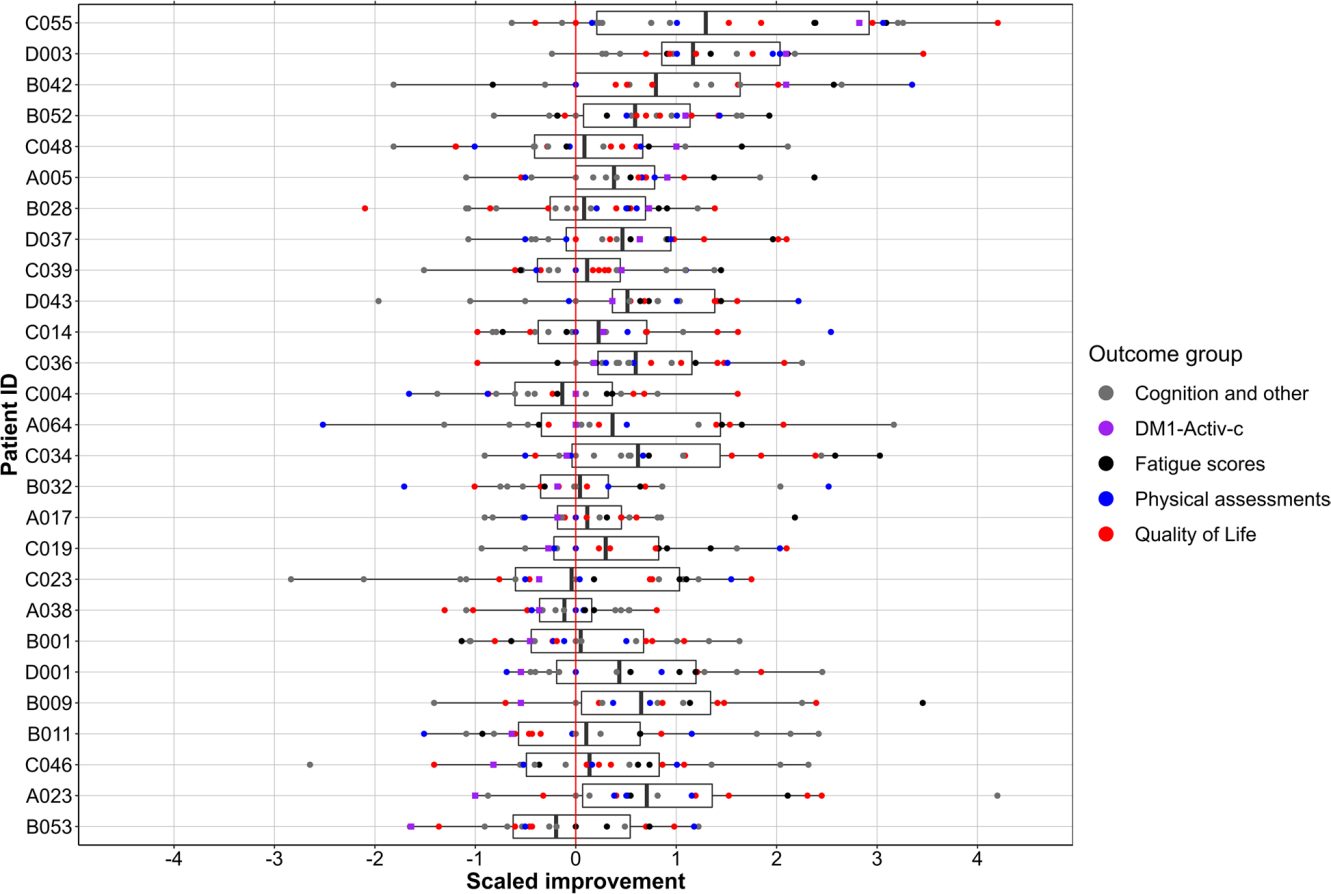
Distribution of changes in outcome measures per patient. Per outcome measure, changes between baseline and 10 months of CBT were scaled by the root mean square. Additionally, for some outcome measures, a sign adjustment was performed so that an increased score is always associated with improved health status. All outcome measures are shown per patient, where patients were ordered along the *y*-axis by their change in DM1-Activ-c scores (purple squares). Boxes enclose the 25th to 75th percentiles, divided by a thick line that represents the mean compound response score. The whiskers represent the lowest/highest value no further than 1.5 times the interquartile range. *Quality of life (red)*: Myotonic Dystrophy Health Index, Individualized Neuromuscular Quality of Life Questionnaire, Adult Social Behavioural Questionnaire, Illness Management Questionnaire, Checklist individual strength — Subscale activity. *Physical assessments (blue)*: Six-Minute Walk Test, BORG Scale, accelometery measures. *Fatigue scores (black)*: Fatigue and Daytime Sleepiness Scale, Checklist Individual Strength — Subscale fatigue, Jacobsen Fatigue Catastrophizing Scale. *Cognition and other (gray)*: Trail Making Test, Stroop Color-Word Interference Test, McGill Pain Questionnaire, Beck Depression Inventory — Fast Screen, Social Support — Discrepancies and Negative Interactions, Apathy Evaluation Scale — Clinical version, Self-Efficacy Scale 28

Samples and metadata used for this study were all gathered during the OPTIMISTIC clinical trial [18]. At the different time points in the trial, blood was drawn and a wide range of clinical outcome measures were recorded. Figure 1 has been generated to illustrate the heterogeneity in changes across all grouped outcome measures, with annotation of all individual outcome measures in the legend. In order to maximize the generalizability of the study findings, the goal of the patient sampling was to obtain a balanced subset of the OPTIMISTIC intervention group (*n*=128). Additionally, by capturing the whole range of therapy responses in a continuous uniform distribution, as assessed by the primary clinical trial outcome DM1-Activ-c [27], strong linear associations between non-responders and responders could be studied. To promote future research, the sampling was furthermore done on the most completely characterized patients. To achieve this, several filter steps have been applied before the random sampling. Patients were selected for which the DM1-Activ-c questionnaire results were available at each time point (*n*=104), with less than 20% missing values for other outcome measures (*n*=81) and without a variant CTG repeat (*n*=74). Homogeneity of baseline disease severity was accounted for by selecting patients that were within one interquartile range (IQR) of the mean for the baseline variables DM1-Activ-c, 6MWT, and CTG-repeat length (*n*=45). One patient was excluded because of polypharmacy (*n*=44). One patient was excluded because of a drop of 57 points of the DM1-Activ-c score between the baseline and 5-month assessments with a subsequent increase of 55 points between the 5- and 10-month assessments (*n*=43).

The distribution of these 43 patients over the clinical sites A, B, C, and D was A12, B11, C16, and D4. Therefore, all patients from site D were selected. For the remaining sites, a stratified random sampling approach was implemented, where patients were randomly sampled from the different sites and a maximum of two patients with the same change in DM1-Activ-c were selected. This process of random selection was repeated until a reasonable site and delta-DM1-Activ-c distribution was achieved, defined as more than 7 patients for sites A, B, and C, resulting in the final selection of 30 patients. Due to the unavailability of samples and unsuccessful RNA sequencing, three patients were later excluded (*n*=27). The final selection featured a site distribution of 5 times center A, 8 B, 10 C, 4 D, and 22 unique changes DM1-Activ-c scores, with no change in the DM1-Activ-c score being present more than twice.

### RNA sequencing

Blood drawn during the OPTIMISTIC trial was collected in Tempus tubes and centrally stored at the New Castle MRC Centre for Rare & Neuromuscular Diseases biobank with strict SOPs and temperature control (−80°C). RNA was locally isolated in Nijmegen using the Tempus Spin RNA Isolation Kit (Applied Biosystems/Thermo Fisher Scientific) according to the manufacturer’s instructions. The concentration and RNA Integrity Number (RIN) were checked using Fragment Analyzer (Thermo Fisher Scientific). The mean RIN value was 8.9 and all were > 7.5. Hemoglobin mRNA was depleted using the Globinclear kit (Thermo Fisher Scientific). Libraries were prepared using NEBNext Ultra II Directional RNA Library Prep Kit (Illumina) according to the manufacturer’s instructions for a polyA mRNA workflow using UMI-indexed adapters. The size distribution (between 300 and 500 bp) was confirmed using Fragment Analyzer. A total of 150-bp paired end sequencing was performed with a NovaSeq6000 machine (Illumina) at a library concentration of 1.1 nM, generating > 30 M read pairs per sample. All raw sequencing data and associated genotype/phenotype/experimental information is stored in the European Genome-phenome Archive (EGA) under controlled access with Dataset ID EGAS00001005830 [28].

### RNA-sequencing primary data analysis

Adapter sequences and low-quality base calls were removed from fastq files using cutadapt 3.4 via TrimGalore 0.6.6 at no other default parameters than the --paired flag [29]. Trimmed fastq files were mapped to the human genome version hg38.95 using STAR 2.7.0 at default parameters and --outSAMtype BAM SortedByCoordinate [30]. After indexing using samtools [31] at default parameters, PCR duplicates were removed from the bam files using umi-tools dedup with the flags --spliced-is-unique, --paired and --output-stats (Additional file 1: Table S1) [32]. Strandedness was verified via RSeQC’s infer_experiment [33]. After indexing the deduplicated bam files, reads were counted for overlap with hg38.95 genes via HTSeq with parameters --format bam --order pos and --stranded=yes [34]. EPIC, quanTIseq, and xCell algorithms were applied to the count tables to verify that the cell type compositions were similar at the two time points [35–37]. GATK HaplotypeCaller and Picard GenotypeConcordance were used to check the correct matching of samples from the same patient [38, 39].

Splice analysis was performed using rMATS v4.1.0 [40] via the same gtf as for STAR/HTSeq with the parameters and flags: -t paired --readLength 150 --variable-read-length --novelSS --libType fr-firststrand --statoff.

### Comparative DM1 datasets

In order to validate our findings, we obtained several external DM1 gene expression datasets of different tissue types (tibialis muscle [11, 41], heart [15, 42], brain [12, 43], and peripheral blood [25, 44]). Additionally, table S5 from Wang et al. was directly obtained from the publication [11]. The dataset EV10 from Signorelli et al. was obtained for the association between gene expression (logFC) and body or performance test in DMD patients [22]. To compare DM1 samples to controls, a two-sided two-sample Wilcoxon test was performed using *row_wilcoxon_twosample* in matrixTests R package on normalized, log-transformed gene counts [45].

### Statistical analysis

All statistical analyses were carried out in R [46]. For gene expression analysis, firstly, genes with low read counts before and after CBT were filtered using edgeR filterByExpr with group = before/after CBT and min.count = 50 [47]. Following, normalized logCPM values and weights were calculated from the filtered read counts with Voom in Limma [48].

To achieve an overarching measure for CBT response, first, the changes for each outcome measure were calculated per patient by subtracting the value after 10 months of CBT from that at baseline. Where applicable, outcome measures were multiplied by −1 in order to always associate positive changes with an improved health status. Using R base *scale*, the changes per outcome measure were then scaled without centering to account for the different scales of the outcome measures. Finally, for each patient, a “Compound Response” score was calculated based on the mean of all scaled outcome measures. Individual contributions towards this compound response score were visualized (Fig. 1). L5ENMO, the mean activity during rest, was a control parameter in OPTIMISTIC and was excluded from this analysis.

We first set out to explore differential gene expression before and after the CBT intervention. Gene expression values from Voom (in logCPM) were separately modeled using mixed effect models with before/after CBT (categorical) as fixed effect and patient identity as random effect (1). Gene weights were also carried over from Voom. This analysis has been implemented using *lme* in the lme4-wrapper lmerTest [49, 50]. lmerTest estimates a *p*-value for the contribution of fixed effects to the model via Satterthwaite’s degrees of freedom method. Parameters of the fits were extracted with R base *summary* and *p*-values were FDR corrected via the Benjamini and Hochberg method with stats *p.adjust* [51].

1 $$Gene\ expression= CBT\left( 0/ 1\right)+\left( 1| Patient\right), gene\ weights$$

In order to study the cohort heterogeneity of gene expression changes, we calculated the logCPM-based difference in gene expression between before and after the intervention for each patient for the 560 genes significantly associated with the CBT predictor of (1) (adjusted *p* < 0.05). Patients and genes with similar expression changes were clustered using the R package heatmap3 based on the complete linkage method for hierarchical clustering, with gene expression values being centered and scaled per gene [52]. Changes in clinical response (DM1-Activ-c, Six-Minute Walk Test (6MWT), and compound response) were visualized using the corrplot function and added to the heatmap [53]. The changes in DM1-Activ-c and 6MWT were scaled using R base *scale* without centering to account for the different scales.

Next, using the same methodology as for the CBT intervention effect, we set out to explore the associations of the different clinical outcome measures and CTG-repeat length with gene expression. For this purpose, we separately modeled gene expression values with either one of the outcome measures or the CTG-repeat length (at the trial start, (2) as fixed effect and patient identity as random effect. The categorical CBT covariate (before/after) was included for each fit to correct for differences between the two time points.

2 $${\displaystyle \begin{array}{c} Gene\ expression= CBT\left(0/1\right)+ CTG\_ repeat\ast +\left(1| Patient\right), gene\ weights\\ {}\ast \textrm{Same}\ \textrm{repeat}\ \textrm{length}\ \textrm{fitted}\ \textrm{for}\ \textrm{both}\ \textrm{T}0\ \textrm{and}\ \textrm{T}10\textrm{M}\end{array}}$$

Analogous to the methodology described for the CTG-repeat association analyses, genes associated with overarching clinical response were identified by fitting separate mixed effect models for each gene with the two fixed effects CBT (categorical) and compound response, as well as patient identity as random effect (3). Notably, the compound response variable has only been fitted for gene expression after the CBT intervention (zero at baseline). As such, the compound response predictor reflects the difference between the two time points that can be attributed towards therapy responsiveness, while accounting for non-therapy-specific differences by including the categorical CBT predictor.

3 $${\displaystyle \begin{array}{c} Gene\ expression= CBT\left(0/1\right)+ Compound\_ Response\left(0/ cont.\right)\ast +\left(1| Patient\right), gene\ weights\\ {}\ast Compound\_ Response\ is\ zero\ at\ T0\ and\ continuous\ at\ T10M\end{array}}$$

Potential biomarker candidates were discovered by intersecting the genes significantly associated with the CTG_repeat predictor from model (2) and the Compound_Response predictor from model (3). Pearson correlation coefficients and the associated nominal *p*-values were calculated between the change in gene expression and the change in clinical score (compound response, delta-DM1-Activ-c) for these potential biomarkers using the corr.test function of the R package “psych” [54].

For the splicing analysis, the PSI values for splice exclusion (SE) events were extracted from the rMATS output and fitted in linear models similar to those for gene expression. Splice events were filtered by excluding exons from the analysis with less than three mapping reads and one junction spanning read in at least 14 samples.

The R package ggplot2 was used for representation in volcano and scatter plots [55]. The R package VennDiagram was used to generate the Venn diagram [56].

### Gene set enrichment analysis

Gene set enrichment analyses have been independently implemented for the gene sets associated with CBT, CTG-repeat length, compound response scores, and the genes significantly associated with both CTG-repeat length and compound response using gProfiler [57]. For the CBT, CTG-repeat length, and compound response-associated genes, the 500 genes with the lowest nominal *p*-values were ordered (decreasing) based on their absolute regression coefficients. Subsequent enrichment analyses were implemented using the R client of gProfiler with the parameter orderd_querey = TRUE against a custom background of 10,292 genes expressed in our samples. Multiple testing correction was based on the default setting “g_SCS.” We tested for enrichment (one-sided) pathways within the WikiPathway database. The gene set associated with both CTG-repeat length and compound response was based on an FDR threshold of 10% for the respective regression coefficients, resulting in a gene set of 311 genes. A regular, non-order weighted ORA (over-representation analysis) analysis was run for this gene set with ordered_quere = FALSE. Similar to the other analyses, one-sided (enrichment) pathway discovery was based on the WikiPathway database with the default setting “g_SCS” to correct for multiple testing. For all enrichment analyses, only significant pathways (*p*-adjusted < 0.05) are reported.

The exact scripts and the resulting datasets of the statistical analyses are available via https://github.com/cmbi/DM1_blood_RNAseq

## Results

### Patient sampling, procedure, and analysis of outcome measures

For the identification of blood biomarkers that are associated with the clinical response to the CBT intervention, 27 patients from the OPTIMISTIC cohort were selected based on a random stratified sampling procedure. These patients reflected a uniform continuous distribution of therapy responses as assessed by the primary trial outcome, the DM1-Activ-c questionnaire.

The sampled set consisted of 14 females and 13 males aged 19–63 years and represented a wide range of CTG-repeat lengths (Additional file 2: Fig. S1). All sampled patients received CBT, and mRNA-sequencing profiles were obtained at baseline and after 10 months of CBT, the primary endpoint of the OPTIMISTIC trial. Large clinical heterogeneity of clinical responses after CBT was observed across all of the different outcome measures. Figure 1 highlights the scaled differences across these outcome measures, color coded into five different groups (cognition and other, DM1-Activ-c, fatigue scores, physical assessments, and quality of life). Because of the large heterogeneity, we defined a compound response score. The compound response score is the mean of all scaled outcome measures (Fig. 1).

### Marked changes in gene expression after CBT

**Fig. 2:**
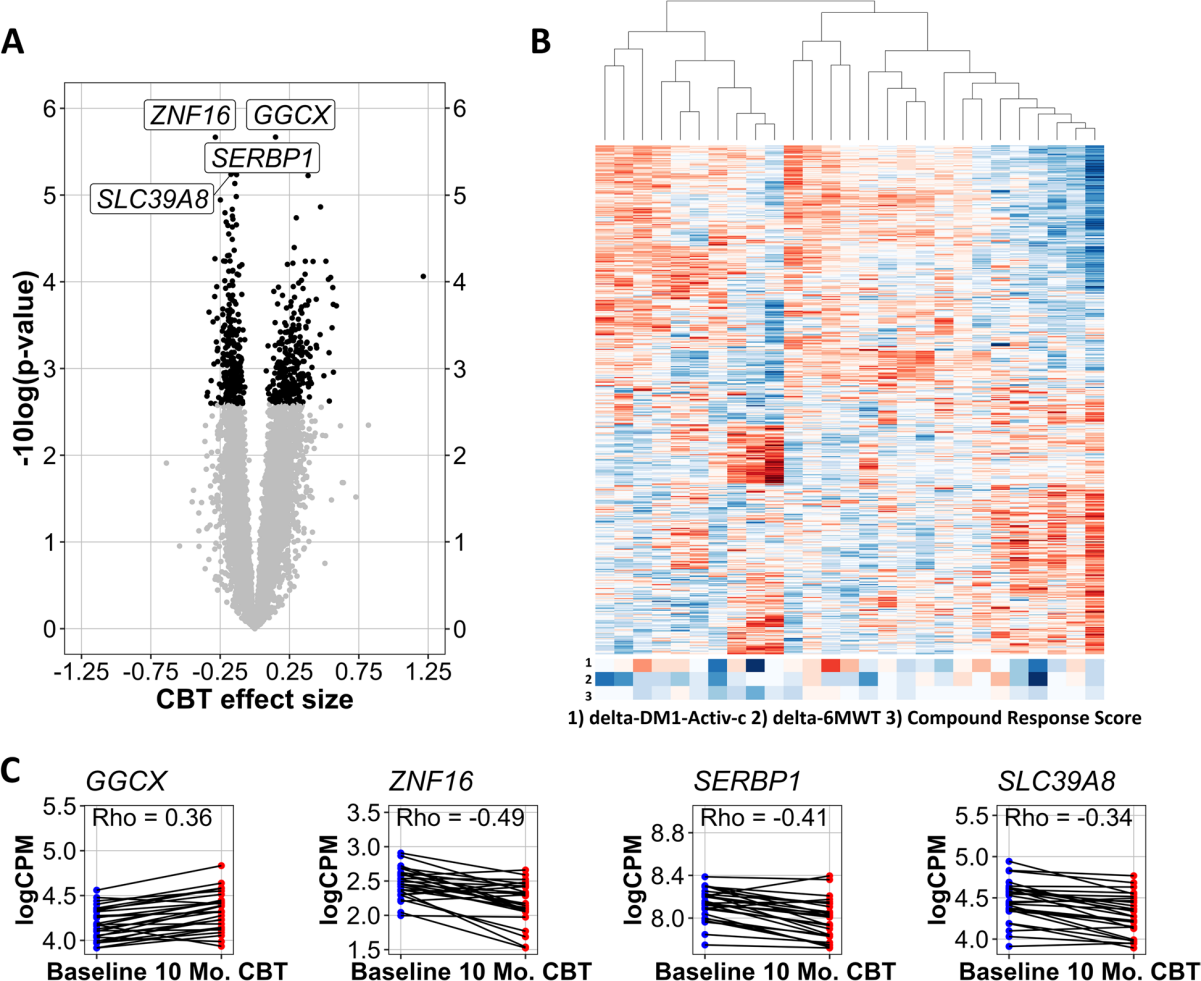
Changes in gene expression after cognitive behavioral therapy. A linear mixed effect model was fitted for each gene, estimating the fixed effect of CBT while accounting for random effects of the individual. The *p*-values for the fixed effect were estimated via Satterthwaite’s degrees of freedom method and FDR corrected. **A** Volcano plot of significance (−log10 of the nominal *p*-value) and the effect size for changed expression after 10 months of CBT. Genes for which the effect size of CBT is significant (FDR < 0.05) are visualized in black. **B** Heatmap of changes in normalized logCPM values between the baseline and the 10-month assessment for the 560 genes significantly associated with the CBT effect size (scores ranging from −3 (dark red) to +3 (dark blue)). Patients and genes were clustered based on the complete linkage method for hierarchical clustering, and values were centered and scaled per gene. For each patient, changes in DM1-Activ-c (delta-DM1-Activ-c) and Six-Minute Walk Test (delta-6MWT) as well as the compound response score were added (scores ranging from −1.6 (dark red) to +2.76 (dark blue)). Delta-DM1-Activ-c and delta-6MWT were scaled by their root mean square. **C** Expression values (logCPM) at baseline (blue) and after CBT (red) of the four genes with the lowest nominal *p*-values from panel **A** including their Pearson correlations

We first studied the molecular changes that occurred after the CBT intervention by comparing the mRNA expression levels in blood before and after the CBT intervention. We found that 560 genes were significantly up- or downregulated after CBT (277 genes down, 283 up, fold changes ranging between 0.64 and 2.35, Fig. 2A). Hierarchical clustering of patients based on the changes in these 560 genes revealed substantial molecular heterogeneity within this 10-month timeframe. There was no evident concordance between the clustering of samples based on changes in gene expression and the changes in DM1-Activ-c, 6MWT, or compound response score (Fig. 2B). The four genes with the lowest *p*-values were *GGCX*, *ZNF16*, *SERBP1*, and *SLC39A8* (Fig. 2C). Biological pathways significantly associated with these genes were limited to an immunological pathway and an electron transport chain in mitochondria (Table 1). *DMPK* expression did not significantly change over the course of the study, nor was it associated with the CTG-repeat length (Additional file 2: Fig. S2).

**Table 1:** Gene set enrichment analysis

Query*	Adjusted ***p***-value**	Term size***	Intersection size****	Term ID	Term name	Intersection with WikiPathway
CBT_ORA	0.014	49	5	WP:WP5039	SARS-CoV-2 innate immunity evasion and cell-specific immune response	*CXCL1,PPBP,PF4,CXCR2,CXCL5*
CBT_ORA	0.037	91	8	WP:WP111	Electron transport chain (OXPHOS system in mitochondria)	*MT-ND3,ATP5F1E,MT-ND1,MT-ATP6,MT-CO2,MT-CO3,MT-ND4,MT-CO1*
CTG_ORA	<0.001	46	10	WP:WP286	IL-3 signaling pathway	*FOS,CSF2RB,GAB2,HCK,LYN,MAPK3,RAF1,STAT3,STAT5B,PIK3CD*
CTG_ORA	0.001	49	10	WP:WP395	IL-4 signaling pathway	*FOS,IRS2,CEBPB,GAB2,NFIL3,MAPK3,STAT6,STAT3,STAT5B,PIK3CD*
CTG_ORA	0.002	26	3	WP:WP4564	Neural crest cell migration during development	*MMP9,EPHB4,FOS*
CTG_ORA	0.003	28	3	WP:WP4565	Neural crest cell migration in cancer	*MMP9,EPHB4,FOS*
CTG_ORA	0.004	29	7	WP:WP3972	PDGFR-beta pathway	*FOS,MAPK3,RAF1,MAP2K4,STAT6,STAT3,STAT5B*
CTG_ORA	0.006	29	2	WP:WP4808	Endochondral ossification with skeletal dysplasias	*MMP9,ALPL*
CTG_ORA	0.006	29	2	WP:WP474	Endochondral osssification	*MMP9,ALPL*
CTG_ORA	0.006	56	2	WP:WP2324	AGE/RAGE pathway	*MMP9,ALPL*
CTG_ORA	0.008	48	10	WP:WP304	Kit receptor signaling pathway	*FOS,GAB2,SH2B2,LYN,MAPK3,RAF1,STAT3,STAT5B,CRK,RPS6KA1*
CTG_ORA	0.011	37	8	WP:WP127	IL-5 signaling pathway	*FOS,CSF2RB,LYN,MAPK3,RAF1,STAT3,STAT5B,RPS6KA1*
CTG_ORA	0.013	31	7	WP:WP313	Signaling of hepatocyte growth factor receptor	*FOS,PTEN,MAPK3,RAF1,STAT3,CRK,PXN*
CTG_ORA	0.015	110	16	WP:WP3929	Chemokine signaling pathway	*CXCR2,TIAM2,HCK,NCF1,LYN,ARRB2,GNB2,PREX1,MAPK3,RAF1,STAT3,STAT5B,CRK,WAS,PXN,PIK3CD*
CTG_ORA	0.031	15	3	WP:WP3599	Transcription factor regulation in adipogenesis	*IRS2,CEBPD,CEBPB*
CTG_CR_ORA	0.002	110	13	WP:WP3929	Chemokine signaling pathway	*WAS,PXN,HCK,MAPK3,PREX1,ARRB2,PIK3R5,VAV1,NCF1,CXCL16,PIK3CD,GNB2,GRB2*
CTG_CR_ORA	0.006	46	8	WP:WP286	IL-3 signaling pathway	*GAB2,CSF2RB,HCK,MAPK3,PTPN6,VAV1,PIK3CD,GRB2*
CTG_CR_ORA	0.009	48	8	WP:WP304	Kit receptor signaling pathway	*GAB2,MAPK3,PTPN6,RPS6KA1,VAV1,SH2B2,JUNB,GRB2*
CTG_CR_ORA	0.016	115	12	WP:WP306	Focal adhesion	*ACTN1,PXN,HCK,MAPK3,CCND2,VASP,TLN1,VAV1,ZYX,ITGA5,PIK3CD,GRB2*
CTG_CR_ORA	0.032	31	6	WP:WP3937	Microglia pathogen phagocytosis pathway	*HCK,PTPN6,VAV1,NCF1,ITGB2,PIK3CD*

*Query refers to the gene sets used. CBT: 560 genes differentially expressed after the intervention; CTG: 608 genes associated with the CTG repeat; CTG_CR: 97 genes associated with both CTG repeat and compound response; *ORA* over-representation analysis

**p-values associated with the finding after correction for multiple testing

***Number of genes in the identified pathway

****Number of genes in the provided gene set overlapping with the pathway

### CTG-repeat length associations reflect molecular dysregulation in blood across different studies

Having a readily accessible fingerprint reflecting the molecular dysregulations associated with DM1 is potentially of high value for both clinical and research settings. To this end, we studied the correlations of blood-based gene expression with the disease-causing CTG-repeat length assessed at the start of the trial. The assessments were based on small pool PCR, *Acil* digestion, and Southern blot, as opposed to (estimations) of CTG-repeat length at birth or disease onset. This was done to minimize potential confounding effects related to the progressive nature of the disease and to assure homogeneity in the measurement methodology.

**Fig. 3:**
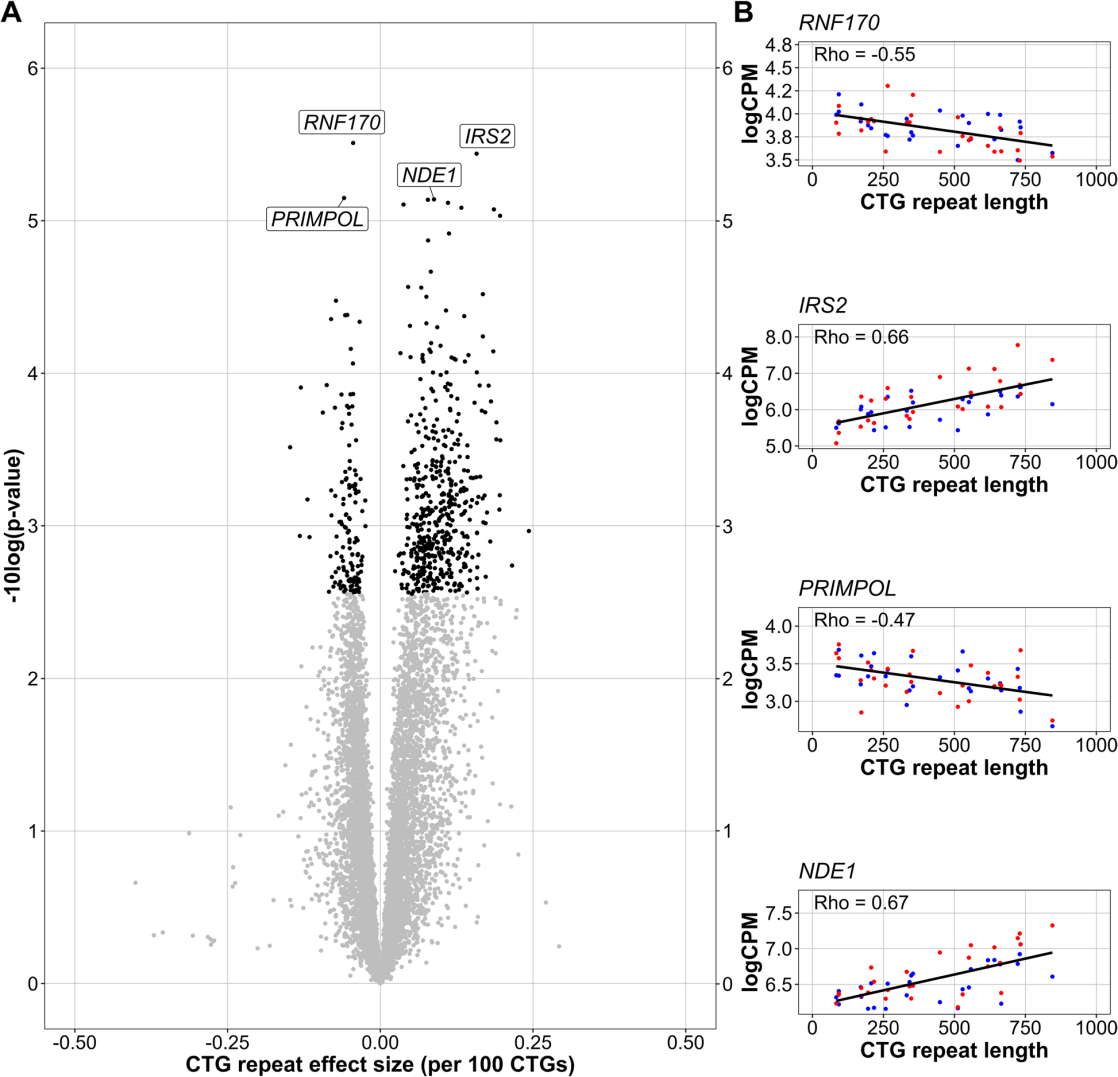
Gene expression levels associated with CTG-repeat length. For each gene, a mixed effect model was fitted with before/after CBT and CTG-repeat length as fixed effects, while accounting for (random) effects of the individual. The *p*-values for the fixed effects were estimated via Satterthwaite’s freedom method and FDR corrected. **A** Volcano plot of significance (−log10 of the nominal *p*-value) and effect size of the CTG-repeat length (per 100 CTGs) on gene expression. Genes for which the effect of CTG-repeat is significant (FDR < 0.05) are visualized in black. **B** For the four genes with the lowest nominal *p*-values from panel **A**, the gene expression values (logCPM) are plotted against the CTG-repeat length. Blue dots represent baseline expression values, and red dots expression values after CBT. The regression line is fitted over all values, independent of the time point. The Pearson correlation coefficients for the association between CTG-repeat length and gene expression are displayed for each gene

Based on this approach, we identified 608 genes significantly associated with the CTG repeat at an FDR cutoff of 5% (474 positively, 134 negatively, fold changes ranging between 0.76 and 1.23 per 100 CTGs, Fig. 3A). The four genes with the lowest *p*-values *RNF170*, *IRS2*, *NDE1*, and *PRIMPOL* showed a clear linear relationship between the CTG-repeat length at baseline and expression values, both before and following the intervention (Fig. 3B). Most enriched pathways were related to immunological processes (IL-3, IL-4, IL-5 signaling; chemokine signaling pathway), yet also pathways related to adipogenesis, hepatocyte signaling, and AGE/RAGE were discovered. Interestingly, the gene *MMP9* was among several of the CTG-repeat-associated pathways.

To replicate and validate that these findings are disease relevant, we compared the CTG-repeat length effect size with effect sizes reflecting the differences in gene expression between DM1 and controls in various published datasets (Additional file 2: Fig. S3). We found a strong correlation with a study that performed mRNA sequencing on DM1 and control blood samples (Pearson rho: 0.59, Additional file 2: Fig. S3A [25]). However, similar correlations were not observed for studies profiling DM1 and control tissues other than blood (heart [15], brain [12], and muscle [11], Additional file 2: Fig. S3B-S3D). Neither were correlations observed with inferred *MBNL* activity or muscle strength measures from another study (Additional file 2: Fig. S3E-S3F [11]). Although no correlations were found with the effect size of blood-based physical test assessments in DMD (Additional file 2: Fig. S3G), the CTG-repeat effect size did correlate well with the effect size of blood-based DMD body measures (Pearson rho: 0.41, Additional file 2: Fig. S3H) [22]. In the latter study, comparisons with physical tests and body measures were independently based on the first principal component of a set of different clinical assessments of DMD patients. Thus, while DM1-related molecular dysregulations in blood can be validated in other studies, even from other neuromuscular disorders, they do not necessarily reflect expression dysregulation observed in other tissues.

Since DM1 is known as a splicing disease, we also studied the association of the CTG-repeat length with alternative splicing events. Here, four events in three genes (*RBM39*, *FLNA*, and *CTSZ*) reached an FDR threshold of <5% (Additional file 2: Fig. S4). Given the limited and small effects observed, we did not further explore these associations.

### Non-significant associations between gene expression and individual clinical outcome measures

Next, we studied phenotype-genotype relationships by estimating the associations of gene expression values with individual clinical outcome measures used in the OPTIMISTIC trial. Here, we found virtually no significant associations between gene expression and clinical outcomes. No significant associations after multiple testing correction were found for the DM1-Activ-c questionnaire (Additional file 2: Fig. S5). The four genes with the lowest nominal *p*-values were *SREBF2*, *ZNF283*, *SF3B3*, and *GSKIP.*

### Significant associations with average clinical response

**Fig. 4:**
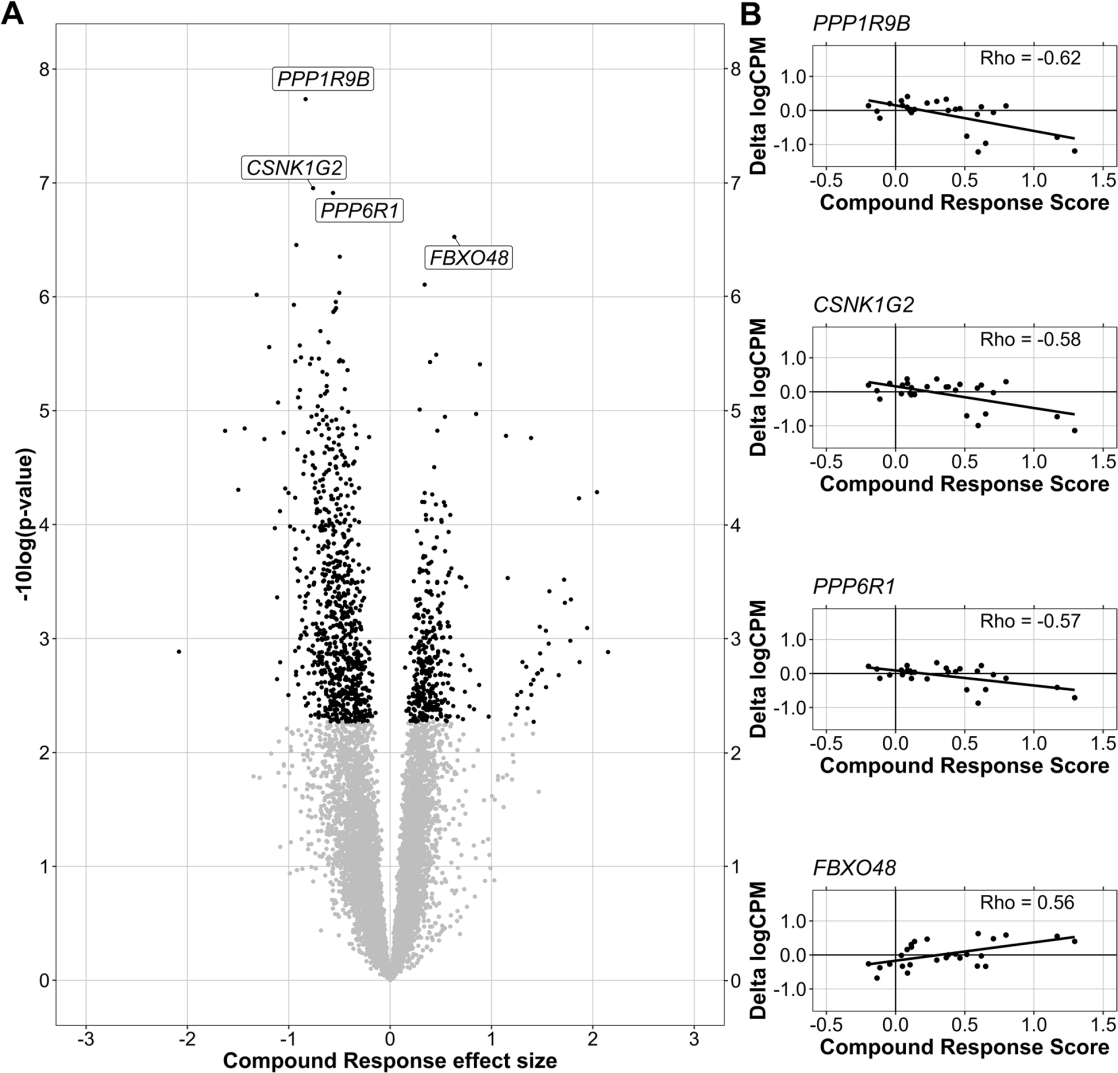
Gene expression association with compound response scores. For each gene, a mixed effect model was fitted with before/after CBT and compound response scores as fixed effects, while accounting for (random) effects of the individual. Compound response scores were fitted for gene expression values after CBT and set to be zero at baseline; the effect size of this covariate therefore expresses changes in gene expression compared to the baseline values that are associated with clinical response. The *p*-values for the fixed effects were estimated via Satterthwaite’s freedom method and FDR corrected. **A** Volcano plot of significance (−log10 of the nominal *p*-value) and the effect size of the compound response score on gene expression. Genes for which the effect size of compound response is significant (FDR < 0.05) are visualized in black. **B** For the four genes with the lowest nominal *p*-values from panel **A**, the changes in gene expression (delta logCPM after-before CBT) are plotted against the compound response scores, including Pearson correlations

To account for the heterogenic changes in individual outcome measures, we calculated a compound CBT response score that reflects the average scaled therapy response of all outcome measures used in OPTIMISTIC (Fig. 1). Noteworthy, similar ranges of change are observed for the variety of outcome measures due to the applied scaling. As such, each outcome measure contributes similarly to the compound response score. The compound response score can be interpreted as an estimate of an overall difference in capacity between the end and the start of the intervention. We were able to identify 1176 genes significantly (FDR < 0.05) associated with the compound response score (384 positive, 792 negative, Fig. 4A). The four hits with the lowest *p*-values (*PPP1R9B*, *CSNK1G2*, *PPP6R1*, *FBXO48*) show a clear linear relationship between changes in gene expression during the trial and the compound response score (Fig. 4B). No enriched pathways were identified for this gene set.

### Clinical improvement linked to the reversal of disease-induced gene expression

**Fig. 5:**
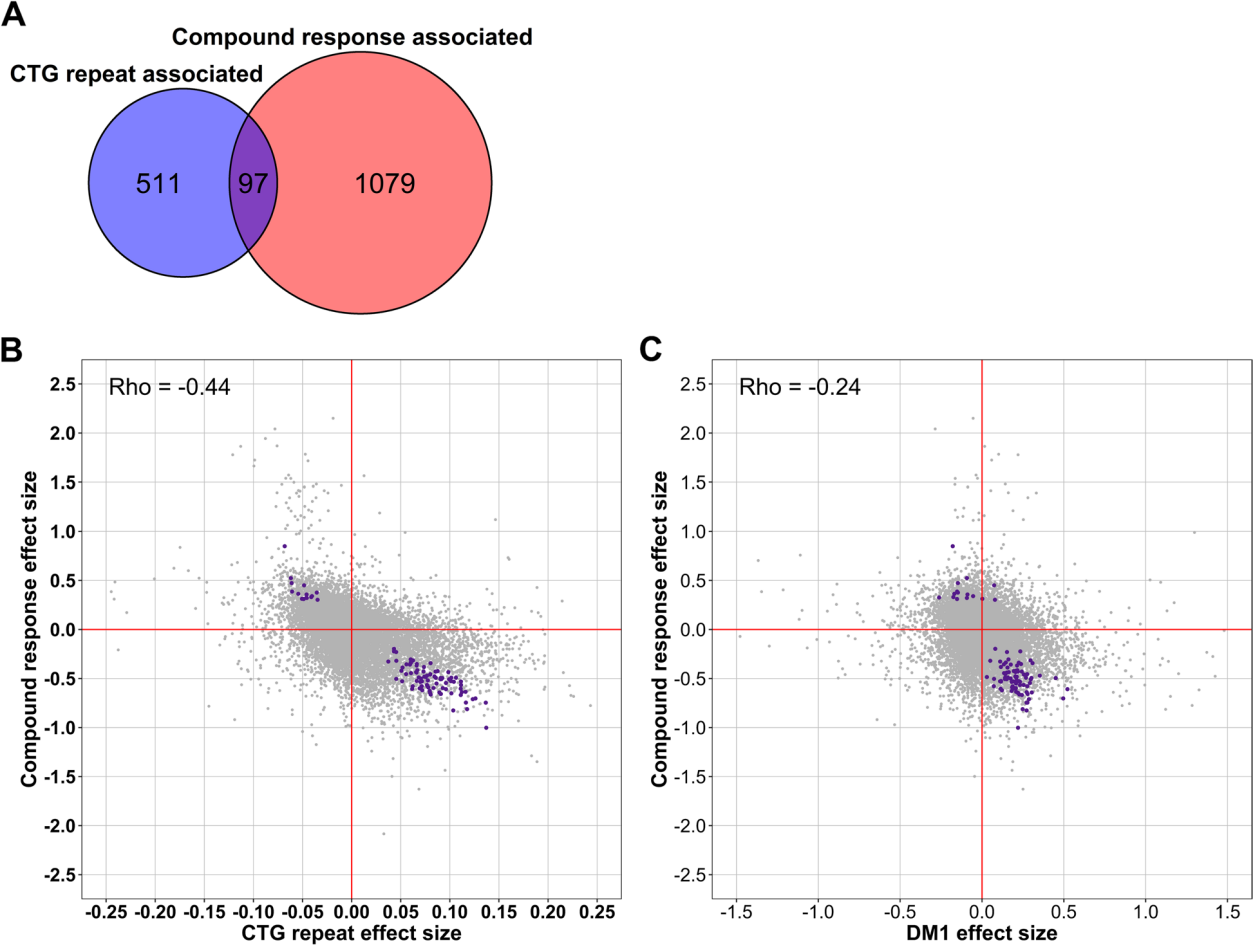
Clinical improvement is linked to normalization of expression of CTG-repeat-associated genes. Linear mixed effect models were fitted for each expressed gene, with CBT as a fixed effect, patient as a random effect, and either CTG repeat or compound response as the predictor. *p*-values for the regression coefficients were estimated via Satterthwaite’s degrees of freedom and considered significant for values smaller than 0.05 after FDR correction. Furthermore, differences in gene expression of blood samples from DM1 patients and controls were calculated based on an external study using a Wilcoxon signed-rank test on normalized, log-transformed gene counts. **A** Venn diagram illustrating the number of significant genes associated with CTG-repeat length and compound response, as well as their overlap (disease-relevant changes). **B** For all expressed genes, the regression coefficients of the compound response scores are plotted against the regression coefficients of the CTG-repeat lengths, including their Pearson correlation. For illustrative purposes, the regression coefficients of the CTG repeat have been multiplied by 100. Furthermore, the *x*-axis has been scaled between −0.25 and 0.25, removing 12 outliers from the figure. Colored in purple are the genes for which both regression coefficients were significant (FDR < 0.05). **C** For all expressed genes, the compound response effect size is plotted against the DM1 effect size based on an external study comparing blood expression profiles from DM1 patients and controls, including their Pearson correlation [25]. For illustrative purposes, the *x*-axis has been scaled between −1.5 and 1.5, removing 6 outliers from the figure. Colored in purple are the same 97 genes as in panel **B**

Since we were able to identify genes significantly associated with both the CTG-repeat length as well as the average clinical response, we were interested in their intersection. Among the significant hits of both analyses, we found an overlap of 97 genes (Fig. 5A). To further investigate this relationship, we plotted the effect size of the compound response score against the effect size of the CTG-repeat length for each expressed gene (Fig. 5B). For the 97 genes significantly associated with both predictors, a remarkable pattern emerged: genes that were lower expressed in patients with a long CTG repeat showed an increase in expression levels in the patients with a good clinical response and vice versa. This anticorrelation pattern was confirmed by analyzing an earlier dataset comparing gene expression in DM1 and control blood samples [25]. Here, the 97 genes showed a similar association with the DM1 phenotype as has been found with the CTG length in our study, confirming that the gene expression of patients with a good CBT response changed into the direction of the levels observed in healthy controls (Fig. 5C). This remarkable relationship could not be explained by possible confounding between CTG-repeat length and compound response, as the compound response effect size is only slightly affected by first regressing out the CTG-repeat length effect (Additional file 2: Fig. S6). The four genes with the lowest *p*-values with both the CTG-repeat length as well as the compound response score (either FDR < 0.025) were *DNAJB12 (CTG Pearson rho=0.49; CRS Pearson rho=−0.43)*, *HDAC5 (CTG rho=0.65; CRS rho=−0.46)*, *TRIM8 (CTG rho=0.59; CRS rho=−0.59)*, and *ZNF22 (CTG rho=−0.52; CRS rho=0.52)*. For 81 of the 97 candidate biomarkers, the Pearson correlations were nominally significant for the change in gene expression and the compound response score. Yet, only 2 of the 97 Pearson correlations were nominally significant for the change in gene expression and the change in DM1-Activ-c scores. Enrichment analysis for these 97 genes resulted in mostly immunological pathways (chemokine and IL-3 signaling, microglia pathogen phagocytosis pathway; Table 1).

In summary, these results suggest that for a subset of genes significantly associated with the biochemical phenotype caused by the CTG-repeat expansion a reversal of disease-induced gene expression occurred in clinical responders. The association with both molecular dysregulation and clinical response makes this subset of genes highly relevant for the discovery of novel therapeutic targets.

## Discussion

The purpose of this study was the identification of DM1-specific therapeutic biomarkers in peripheral blood. The multisystem nature of DM1 is known to be reflected by laboratory abnormalities of peripheral blood, making it together with its accessibility a promising tissue for biomarker studies in this disease [26]. Hence, we used blood samples of 27 DM1 patients from the OPTIMISTIC cohort to study the associations of gene expression with disease severity as well as the response towards the CBT intervention. In an effort to fairly represent the whole OPTIMISTIC cohort and to facilitate the generalizability of the study findings, a stratified random sampling procedure was implemented which resulted in a balanced patient cohort with respect to age, CTG-repeat length, sex, therapy response, and clinical site distributions. Patients with an interrupted CTG repeat were excluded in order to limit molecular heterogeneity induced by slower disease progression rates [5]. Nonetheless, we identified substantial heterogeneity in molecular expression profile changes after the 10-month CBT intervention. Furthermore, we identified gene sets that were significantly associated with the disease-causing CTG repeat as well as with the average response towards the CBT intervention across different clinical outcome measures. Most interestingly, an overlap of 97 genes among these latter two gene sets has been identified, showing a clear trend of more normal expression levels in clinical responders. Based on these different gene sets, several biological pathways associated with DM1 have been discovered, as well as specific genes/gene families with ties to neuro(-muscular) disorders.

The OPTIMISTIC study has shown that DM1 patients significantly improve in their capacity for activity and social participation after the CBT intervention [18]. It was furthermore hypothesized that CBT may directly or indirectly improve other biological systems affected by the disease. This hypothesis has been confirmed for muscles of the lower extremity, showing an increase in cross-sectional area as a result of the intervention [58]. Here, we set out to further explore this hypothesis by investigating changes on the molecular level. These changes may be the result of increased physical activity, which has been linked to differences in gene expression in previous studies [59], but may also be a more direct effect of the psychotherapeutic CBT intervention [60].

In line with the results of an earlier study, we have illustrated that the clinical response towards the CBT intervention was rather heterogenous [21]. A novel addition to this finding was the illustration that this heterogeneity extends towards changes in molecular profiles within a 10-month timeframe. Importantly, this heterogeneity could not be explained by changes in the cellular composition of the blood samples between the two time points, as the similarity of cell type composition has been verified. Additionally, this heterogeneity could not be explained by changes of different outcome measures such as the DM1-Activ-c, 6MWT, or compound response. While the CBT intervention likely played a part in this heterogeneity, the magnitude of this contribution could not be assessed due to the lack of a control group. Other factors, such as aging or seasonal effects, may also have contributed to this finding.

Across the different gene sets identified in this study, several of the genes with the lowest *p*-values (*SLC39A8*, *IRS2*, *FBXO48*) and one WikiPathway (transcription factor regulation in adipogenesis) were associated with insulin signaling or more broadly related to metabolism/adipogenesis [61–63]. Dysregulation of insulin signaling has been linked to clinical features of DM1 and is an actively ongoing field of investigation [64]. Aberrant insulin signaling has also been found in other diseases of the nervous system such as depression, with indirect improvements being observed after CBT [65]. Interestingly, the anti-diabetic drug metformin has been shown to improve the mobility of DM1 patients with effect sizes of the 6MWT comparable to those observed in the OPTIMISTIC study [66]. With increasing therapeutic interest in this area, our findings suggest that disease-relevant insulin signaling can be studied on a molecular level in blood samples, highlighting the utility of peripheral blood in this setting.

Similarly, across most of the gene sets, we identified several WikiPathways associated with immunological functions (cell-specific immune response, chemokine signaling pathway, IL-3, 4, and 5 signaling). While this may be in part due to a bias introduced by the profiled tissue, the immune system likely plays a role in the DM1 pathophysiology like for many other chronic diseases [67]. As such, blood sample-based immunology studies may be an interesting field of future investigation.

The intersection of the genes significantly associated with the disease-causing CTG repeat, as well as the average CBT response across different outcome measures, revealed a subset of 97 genes. These genes are of particular interest for the identification of therapeutic biomarkers, as their disease association has been confirmed in an external dataset and they showed normalization of expression levels in clinical responders. Among the genes with the lowest *p*-values associated to both CTG-repeat length as well as CBT response were *HDAC5*, *DNAJB12*, and *TRIM8.* In total, the subset of 97 genes consisted of two HDACs (histone deacetylases, *HDAC5*, *HDAC7*). HDACs play an important role in transcriptional regulation and compounds that inhibit HDAC enzymes are being studied for their potential effect on a range of human diseases, including neurological disorders [68]. The DNAJB12 protein is a member of the heat shock protein family, with some evidence supporting positive effects of their induction for muscular dystrophy and other muscle wasting conditions [69]. The TRIM family protein TRIM72 has been shown to be an essential component of the cellular membrane repair in muscles, with evidence supporting some positive effects in mouse models of muscular dystrophy [70]. Authors of the same study suggest the potential of other TRIM family members as potential targets in similar disease states, which may support the further investigation of *TRIM8* in DM1. Although mostly associated with therapy response, *RARA* and *RXRA* were also among the overlapping 97 genes. Stimulating retinoic acid signaling has been linked to muscle regeneration in mouse models via increased proliferation of fibro/adipogenic progenitor cells, highlighting the relevance of this pathway as another potentially DM1-relevant drug target [71]. Taken together, these findings confirm the value of whole blood-based expression profiling for the discovery of therapeutic biomarkers in DM1.

Interestingly, the genes significantly associated with the CTG repeat showed a moderate correlation with the genes associated with DMD body measurements of an external study. We hypothesize that these body measures are likely also correlated with age, which in turn reflects disease progression. This suggests that some of our results may therefore not be DM1 specific, but rather reflect non-specific molecular dysregulations shared across different (neuromuscular) disorders. This hypothesis is supported by the significant association between the CTG repeat with *MMP9*, which is known to be a non-specific biomarker that has for instance been linked to cardiac remodeling after myocardial infarction, inflammation, and DMD [72, 73]. We therefore deem further exploration of shared dysregulations as highly valuable, as this may lead to the discovery of therapeutic targets relevant to a variety of diseases.

Although DM1 is known as an alternative splicing disease, only four splice events have been significantly linked to the disease-causing CTG repeat in this study. This may be the result of relatively low *DMPK* expression in blood [74] and is in line with the absence of strong splice aberrations in blood from DM1 patients compared to controls [25]. *DMPK’s* low expression in blood cells may also explain the lack of concordance between our disease severity-associated gene expression differences observed in blood with gene expression differences observed in the muscle and brain. So, while whole blood-based transcription profiling can identify disease-relevant molecular dysregulations, these dysregulations do likely not fully reflect the dysregulations observed in other tissue types. Yet, we found a high correlation of the CTG-repeat effect size with the DM1 phenotype effect size of a different blood-based study, as well as with a principal component derived body measure association of a DMD-based study. While the former validates our findings, the latter suggests the possibility of shared, disease-relevant, dysregulations across different neuro(muscular) disorders detectable in peripheral blood. If true, associated pathways might reveal highly interesting targets for drug discovery, as they may have a positive influence on multiple diseases.

### Limitations of this study

To find disease-relevant gene expression in blood, we searched for linear associations with the disease-causing CTG-repeat length. While the CTG-repeat length is thought to be the main driver of molecular dysregulation, associations between the progenitor allele length of the CTG repeat with several clinical outcome measures, including DM1-Activ-c and 6MWT, have been found to be only weak-moderate [13]. In line with the previously published challenges to directly relate gene expression to clinical phenotypes, we were not able to find significant, direct associations between clinical outcome measures and gene expression. Still, among the genes with the lowest *p*-values for the DM1-Activ-c questionnaire was *GSKIP*, a gene encoding for an inhibitor protein of the known DM1 drug target GSK3-β [75–77]. Given the biological relevance of this finding, we deem it likely that the current study design was underpowered to study the association of gene expression with individual clinical outcome measures, especially when taking clinical and molecular heterogeneity into account.

The clinical heterogeneity in therapy response may in part be explained by the personalized nature of the CBT intervention, with therapy foci being tailored towards the needs and wishes of the individual patient. As a consequence, one might expect different outcome measures to be more appropriate for CBT efficacy assessments for different patients. Yet, the identification of molecular signatures associated with therapy response necessitates the use of the same clinical outcome measure. For this reason, and to average out some of the uncertainty inherently associated with the recording of the different outcome measures, we settled on the use of the compound response score. While the scaling assured a more or less equal contribution of each outcome measure to this score, we acknowledge that this combined score is biased by the outcome measures that were used in OPTIMISTIC.

Even though we statistically corrected for non-specific molecular changes between the two time points, the lack of RNA-seq profiles from the OPTIMISTIC control arm makes it difficult to state with certainty that the observed molecular changes are due to the therapy itself. However, this does not discount their value as potential therapeutic targets, as they are, regardless of the mediation factor, significantly associated with improved clinical status. Moreover, for this reason, we deemed studying the RNA-seq profiles of the OPTIMISTIC control arm to be less valuable, as these patients did not significantly clinically declined over the 10-month timeframe [18].

## Conclusions

Starting from DM1-specific disease determinants, the OPTIMISTIC study has shown that patient-tailored CBT can increase the health status of DM1 patients by improving social participation and activity. It was furthermore hypothesized that the CBT intervention positively challenges the biological system, which has already been confirmed by increased cross-sectional area for muscles of the lower extremities. Making use of the clinical heterogeneity in therapy response, we here additionally confirmed disease-relevant molecular changes in peripheral blood. Not only do our results highlight the utility of peripheral blood to study the multisystem nature of the disease, but also generated the foundation for an upcoming, multi-omics-based drug repurposing study.

## Supplementary Information

**Additional file 1: Table S1.** PCR duplicates.

**Additional file 2: Figure S1.** Patient characteristics. Box-plots illustrating the distribution of age (A) and CTG-repeat length (B) of the 27 patients at baseline, separated by sex. C) Box-plots illustrating the change in DM1-Activ-c score after the intervention versus before, as expressed in Delta DM1-Activ-c scores, separated by sex. **Figure S2.** *DMPK* expression. Panel A shows the expression values of *DMPK* in logCPM before and after the CBT intervention for all 27 patients. Panel B shows the association between the CTG repeat length and the change in *DMPK* expression, as calculated by expression levels after the intervention minus the expression levels at the start of the trial. In addition, the Pearson correlation of this association is shown. **Figure S3.** Comparison of gene expression associated to DM1 in other studies with that to the CTG repeat in this study. The mean differences in normalized gene counts were calculated between DM1 and control samples for four studies comparing blood (A (25, 44)), heart (B (15, 42)), brain (C (12, 43)) and tibialis muscle (D, (11, 41)) and plotted against the effect sizes for the CTG-repeat in this study (ReCognitION) for genes that were measured in both studies. In E and F, the correlation of expression to the inferred MBNL activity and muscle strength(11), was compared to our effects for the CTG-repeat. In G and H, gene expression associations in blood of the results of physical tests and several body measurements for Duchenne muscular dystrophy (DMD) patients (22) are compared to CTG-repeat associations from our study. In (22), associations with physical tests and body measurements were based on the first principal component over associated measures, each reflecting respectively 78% and 70% of the associated measurements variances. Depicted on the top left in each graph is the Pearson correlation coefficient for the plotted values with the associated *p*-values. **Figure S4.** Splice exclusion and CTG-repeat length. PSI values for splice exclusion events were determined using rMATS [. For each of the PSI values a linear mixed effect model was fitted with the modal CTG repeat length as covariate and patient as random effect. A) Volcano plot of significance (-log 10 of the nominal p-values of the modal CTG effect size) and the CTG effect size for the PSI values. Significant results after FDR correction (*p* < 0.05) are marked in black. B) For the four PSI values with the lowest nominal *p*-values from A, the PSI values are plotted against the modal CTG repeat lengths before (blue) and after the CBT intervention (red) including the Pearson correlation coefficients. **Figure S5.** Gene expression association with DM1-Activ-c. For each gene a mixed effect model was fitted with before/after CBT and DM1-Activ-c scores as fixed effects, while accounting for (random) effects of the individual. The p-values for the fixed effects were estimated via Satterthwaite’s freedom method and FDR corrected. A) Volcano plot of the significance (-10log of the nominal p-value) and effect size of the DM1-Activ-c scores on gene expression. B) For the four genes with the lowest nominal p-values from panel A, the DM1-Activ-c scores are plotted against the gene expression values (logCPM). Blue dots represent baseline values, red dots values after CBT. The regression line indicates the linear association independent of the timepoints. Similarly, the Pearson correlation coefficients shown are independent of the timepoints. **Figure S6.** Shared explained variance among CTG-repeat and Compound Response predictors. To assess the overlap in gene expression level variance explained by the CTG-repeat length and the Compound Response score, the Compound Response score was fitted on the residuals of the CTG-repeat length as fixed effect, while accounting for random effects of the patient. A) The effect sizes of the Compound Response score as estimated on the CTG-repeat model residuals are plotted against the effect sizes Compound Response scores as presented in this study. The Rho score reflects the Pearson correlation coefficient. B) Analogous to Figure 5B, the Compound Response score effect size as estimated on the CTG-repeat model residuals are plotted against the CTG repeat effect size size as estimated on the CTG-repeat model residuals are plotted against the CTG repeat effect size scaled between -0.25 and 0.25, resulting in the removal of 12 outliers. Colored in purple are the same 97 genes as in Figure 5B.

## Data Availability

All raw sequencing data and associated genotype/phenotype/experimental information is stored in the European Genome-phenome Archive (EGA) under controlled access with Dataset ID EGAS00001005830 [28].

## Abbreviations

6MWT: Six-Minute Walk Test
CBT: Cognitive behavioral therapy
CELF: CUGBP Elav-like families
CPM: Counts per million reads mapped
DM1: Myotonic dystrophy type 1
DMD: Duchenne muscular dystrophy
DMPK: DM1 protein kinase
EGA: European Genome-phenome Archive
FC: Fold change
FDR: False discovery rate
HDACs: Histone deacetylases
MBNL: Muscleblind-like families
SE: Splice exclusion
UTR: Untranslated region

## References

[1] Meola G, Cardani R (2015). Myotonic dystrophies: an update on clinical aspects, genetic, pathology, and molecular pathomechanisms. Biochim Biophys Acta Mol basis Dis.

[2] Brook JD, McCurrach ME, Harley HG, Buckler AJ, Church D, Aburatani H (1992). Molecular basis of myotonic dystrophy: expansion of a trinucleotide (CTG) repeat at the 3′ end of a transcript encoding a protein kinase family member. Cell..

[3] Mahadevan M, Tsilfidis C, Sabourin L, Shutler G, Amemiya C, Jansen G (1992). Myotonic dystrophy mutation: an unstable CTG repeat in the 3′ untranslated region of the gene. Science..

[4] Fu YH, Pizzuti A, Fenwick RG, King J, Rajnarayan S, Dunne PW (1992). An unstable triplet repeat in a gene related to myotonic muscular dystrophy. Science..

[5] Cumming SA, Hamilton MJ, Robb Y, Gregory H, McWilliam C, Cooper A (2018). De novo repeat interruptions are associated with reduced somatic instability and mild or absent clinical features in myotonic dystrophy type 1. Eur J Hum Genet.

[6] Napierała M, Krzyzosiak WJ (1997). CUG repeats present in myotonin kinase RNA form metastable “slippery” hairpins. J Biol Chem.

[7] Fardaei M, Rogers MT, Thorpe HM, Larkin K, Hamshere MG, Harper PS (2002). Three proteins, MBNL, MBLL and MBXL, co-localize in vivo with nuclear foci of expanded-repeat transcripts in DM1 and DM2 cells. Hum Mol Genet.

[8] Warf MB, Berglund JA (2007). MBNL binds similar RNA structures in the CUG repeats of myotonic dystrophy and its pre-mRNA substrate cardiac troponin T. RNA..

[9] van Cruchten RTP, Wieringa B, Wansink DG (2019). Expanded CUG repeats in DMPK transcripts adopt diverse hairpin conformations without influencing the structure of the flanking sequences. RNA..

[10] Nakamori M, Sobczak K, Puwanant A, Welle S, Eichinger K, Pandya S (2013). Splicing biomarkers of disease severity in myotonic dystrophy. Ann Neurol.

[11] Wang ET, Treacy D, Eichinger K, Struck A, Estabrook J, Wang TT (2018). Transcriptome alterations in myotonic dystrophy skeletal muscle and heart. Hum Mol Genet.

[12] Otero BA, Poukalov K, Hildebrandt RP, Thornton CA, Jinnai K, Fujimura H, et al. Transcriptome alterations in myotonic dystrophy frontal cortex. Cell Rep. 2021;34(3):108634. 10.1016/j.celrep.2020.108634 PMC9272850 33472074

[13] Cumming SA, Jimenez-Moreno C, Okkersen K, Wenninger S, Daidj F, Hogarth F (2019). Genetic determinants of disease severity in the myotonic dystrophy type 1 OPTIMISTIC cohort. Neurology..

[14] Mankodi A, Takahashi MP, Jiang H, Beck CL, Bowers WJ, Moxley RT (2002). Expanded CUG repeats trigger aberrant splicing of ClC-1 chloride channel pre-mRNA and hyperexcitability of skeletal muscle in myotonic dystrophy. Mol Cell.

[15] Freyermuth F, Rau F, Kokunai Y, Linke T, Sellier C, Nakamori M (2016). Splicing misregulation of SCN5A contributes to cardiac-conduction delay and heart arrhythmia in myotonic dystrophy. Nat Commun.

[16] Savkur RS, Philips AV, Cooper TA (2001). Aberrant regulation of insulin receptor alternative splicing is associated with insulin resistance in myotonic dystrophy. Nat Genet.

[17] Roussel M-P, Morin M, Gagnon C, Duchesne E (2019). What is known about the effects of exercise or training to reduce skeletal muscle impairments of patients with myotonic dystrophy type 1? A scoping review. BMC Musculoskelet Disord.

[18] Okkersen K, Jimenez-Moreno C, Wenninger S, Daidj F, Glennon J, Cumming S (2018). Cognitive behavioural therapy with optional graded exercise therapy in patients with severe fatigue with myotonic dystrophy type 1: a multicentre, single-blind, randomised trial. Lancet Neurol.

[19] Manta A, Stouth DW, Xhuti D, Chi L, Rebalka IA, Kalmar JM (2019). Chronic exercise mitigates disease mechanisms and improves muscle function in myotonic dystrophy type 1 mice. J Physiol.

[20] Hu N, Kim E, Antoury L, Li J, González-Pérez P, Rutkove SB (2020). Antisense oligonucleotide and adjuvant exercise therapy reverse fatigue in old mice with myotonic dystrophy. Mol Ther Nucleic Acids.

[21] van As D, Okkersen K, Bassez G, Schoser B, Lochmüller H, Glennon JC (2021). Clinical outcome evaluations and CBT response prediction in myotonic dystrophy. J Neuromuscul Dis.

[22] Signorelli M, Ebrahimpoor M, Veth O, Hettne K, Verwey N, García-Rodríguez R (2021). Peripheral blood transcriptome profiling enables monitoring disease progression in dystrophic mice and patients. EMBO Mol Med.

[23] Byrne LM, Rodrigues FB, Blennow K, Durr A, Leavitt BR, Roos RAC (2017). Neurofilament light protein in blood as a potential biomarker of neurodegeneration in Huntington’s disease: a retrospective cohort analysis. Lancet Neurol.

[24] Spijker S, Van Zanten JS, De Jong S, Penninx BWJH, Van Dyck R, Zitman FG (2010). Stimulated gene expression profiles as a blood marker of major depressive disorder. Biol Psychiatry.

[25] Sznajder ŁJ, Scotti MM, Shin J, Taylor K, Ivankovic F, Nutter CA, et al. Loss of MBNL1 induces RNA misprocessing in the thymus and peripheral blood. Nat Commun. 2020;11(1):2022. 10.1038/s41467-020-15962-x PMC7181699 32332745

[26] Heatwole CR, Miller J, Martens B, Moxley RT (2006). Laboratory abnormalities in ambulatory patients with myotonic dystrophy type 1. Arch Neurol.

[27] Hermans MCE, Hoeijmakers JGJ, Faber CG, Merkies ISJ. Reconstructing the Rasch-built myotonic dystrophy type 1 activity and participation scale. PLoS One. 2015;10(10):e0139944. 10.1371/journal.pone.0139944 PMC4618741 26484877

[28] Full blood mRNA sequencing of myotonic dystrophy type 1 patients after cognitive behavioural therapy. EGA-Archive. https://identifiers.org/ega.dataset:EGAD00001005830. Accessed 01 May 2022.

[29] Martin M. Cutadapt removes adapter sequences from high-throughput sequencing reads. EMBnet.journal. 2011;17(1):10–2.

[30] Dobin A, Davis CA, Schlesinger F, Drenkow J, Zaleski C, Jha S (2013). STAR: ultrafast universal RNA-seq aligner. Bioinformatics..

[31] Li H, Handsaker B, Wysoker A, Fennell T, Ruan J, Homer N (2009). The sequence alignment/map format and SAMtools. Bioinformatics..

[32] Smith T, Heger A, Sudbery I (2017). UMI-tools: modeling sequencing errors in unique molecular identifiers to improve quantification accuracy. Genome Res.

[33] Wang L, Wang S, Li W (2012). RSeQC: quality control of RNA-seq experiments. Bioinformatics..

[34] Anders S, Pyl PT, Huber W (2015). HTSeq-a Python framework to work with high-throughput sequencing data. Bioinformatics..

[35] Racle J, de Jonge K, Baumgaertner P, Speiser DE, Gfeller D (2017). Simultaneous enumeration of cancer and immune cell types from bulk tumor gene expression data. Elife.

[36] Finotello F, Mayer C, Plattner C, Laschober G, Rieder D, Hackl H (2019). Molecular and pharmacological modulators of the tumor immune contexture revealed by deconvolution of RNA-seq data. Genome Med.

[37] Aran D, Hu Z, Butte AJ (2017). xCell: digitally portraying the tissue cellular heterogeneity landscape. Genome Biol.

[38] Poplin R, Ruano-Rubio V, DePristo MA, Fennell TJ, Carneiro MO, Van der Auwera GA, et al. Scaling accurate genetic variant discovery to tens of thousands of samples [Internet]. bioRxiv [Preprint]. 2017 [cited 2021 Sep 01]. 10.1101/201178.

[39] Broad Institute. Picard toolkit. GitHub repository. http://broadinstitute.github.io/picard/. Accessed 01 May 2021.

[40] Shen S, Park JW, Lu ZX, Lin L, Henry MD, Wu YN (2014). rMATS: robust and flexible detection of differential alternative splicing from replicate RNA-Seq data. Proc Natl Acad Sci U S A.

[41] Wang ET, Treacy D, Wang TT, Estabrook J, Day J, Brook D, et al. Transcriptional profiling of myotonic dystrophy muscle and heart. Gene Expression Omnibus. 2016. https://identifiers.org/geo:GSE86356. Accessed 01 Feb 2021.

[42] Charlet-Berguerand N, Takahashi M, Jost B. RNA sequencing of heart samples of myotonic dystrophic (DM1) patients. Gene Expression Omnibus. 2015. https://identifiers.org/geo:GSE67812. Accessed 01 Feb 2021.

[43] Otero BA, Poukalov K, Hildebrandt RP, Thornton CA, Jinnai K, Fujimara H, et al. Transcriptome alterations in myotonic dystrophy frontal cortex. Gene Expression Omnibus. 2020. https://identifiers.org/geo:GSE157428. Accessed 01 Feb 2021. 10.1016/j.celrep.2020.108634 PMC9272850 33472074

[44] Sznajder ŁJ, Scotti MM, Shin J, Taylor K, Ivankovic F, Nutter CA, et al. Loss of MBNL1 induces RNA mis-processing in the thymus and peripheral blood. Gene Expression Omnibus. 2020. https://identifiers.org/geo:GSE138691. Accessed 01 Feb 2021. 10.1038/s41467-020-15962-x PMC7181699 32332745

[45] Schwender H, Müller T. Computing thousands of test statistics simultaneously in R. Stat. Comput Graph. 2007;18(1):5–11.

[46] Team RC (2019). R: a language and environment for statistical computing.

[47] Robinson MD, McCarthy DJ, Smyth GK (2010). edgeR: a Bioconductor package for differential expression analysis of digital gene expression data. Bioinformatics..

[48] Ritchie ME, Phipson B, Wu D, Hu Y, Law CW, Shi W (2015). limma powers differential expression analyses for RNA-sequencing and microarray studies. Nucleic Acids Res.

[49] Bates D, Mächler M, Bolker B, Walker S (2015). Fitting linear mixed-effects models using lme4. J Stat Softw.

[50] Kuznetsova A, Brockhoff PB, Christensen RHB (2017). lmerTest package: tests in linear mixed effects models. J Stat Softw.

[51] Benjamini Y, Hochberg Y (1995). Controlling the false discovery rate: a practical and powerful approach to multiple testing. J R Stat Soc Ser B.

[52] Zhao S, Guo Y, Sheng Q, Shyr Y (2014). Heatmap3: an improved heatmap package with more powerful and convenient features. BMC Bioinforma.

[53] Wei T, Simko V, Levy M, Xie Y, Jin Y, Zemla J (2017). R package “corrplot”: visualization of a correlation matrix. Statistician..

[54] Revelle W. psych: Procedures for Psychological, Psychometric, and Personality Research. Northwestern University, Evanston, Illinois. R package. https://CRAN.R-project.org/package=psych. Accessed 01 May 2021.

[55] Wickham H (2016). ggplot2: elegant graphics for data analysis.

[56] Chen H, Boutros PC (2011). VennDiagram: a package for the generation of highly-customizable Venn and Euler diagrams in R. BMC Bioinformatics.

[57] Raudvere U, Kolberg L, Kuzmin I, Arak T, Adler P, Peterson H (2019). G:Profiler: a web server for functional enrichment analysis and conversions of gene lists (2019 update). Nucleic Acids Res.

[58] Heskamp L, Okkersen K, van Nimwegen MV, Ploegmakers MJ, Bassez G, Deux JF (2020). Quantitative muscle MRI depicts increased muscle mass after a behavioral change in myotonic dystrophy type 1. Radiology.

[59] Grazioli E, Dimauro I, Mercatelli N, Wang G, Pitsiladis Y, Di Luigi L (2017). Physical activity in the prevention of human diseases: role of epigenetic modifications. BMC Genomics.

[60] Jiménez JP, Botto A, Herrera L, Leighton C, Rossi JL, Quevedo Y (2018). Psychotherapy and genetic neuroscience: an emerging dialog. Front Genet.

[61] Li D, Achkar JP, Haritunians T, Jacobs JP, Hui KY, D’Amato M (2016). A pleiotropic missense variant in SLC39A8 is associated with Crohn’s disease and human gut microbiome composition. Gastroenterology..

[62] Brady MJ (2004). IRS2 takes center stage in the development of type 2 diabetes. J Clin Invest.

[63] Liu Y, Jurczak MJ, Lear TB, Lin B, Larsen MB, Kennerdell JR (2021). A Fbxo48 inhibitor prevents pAMPKα degradation and ameliorates insulin resistance. Nat Chem Biol.

[64] Nieuwenhuis S, Okkersen K, Widomska J, Blom P, ‘t Hoen PAC, van Engelen B, et al. Insulin signaling as a key moderator in myotonic dystrophy type 1. Front Neurol. 2019;10:1229. 10.3389/fneur.2019.01229 PMC6901991 31849810

[65] Gulley LD, Shomaker LB, Kelly NR, Chen KY, Stice E, Olsen CH (2019). Indirect effects of a cognitive-behavioral intervention on adolescent weight and insulin resistance through decreasing depression in a randomized controlled trial. J Pediatr Psychol.

[66] Bassez G, Audureau E, Hogrel JY, Arrouasse R, Baghdoyan S, Bhugaloo H (2018). Improved mobility with metformin in patients with myotonic dystrophy type 1: a randomized controlled trial. Brain..

[67] Zhong J, Shi G (2019). Editorial: Regulation of inflammation in chronic disease. Front Immunol.

[68] Gottesfeld JM, Pandolfo M (2009). Development of histone deacetylase inhibitors as therapeutics for neurological disease. Future Neurol.

[69] Thakur SS, Swiderski K, Ryall JG, Lynch GS. Therapeutic potential of heat shock protein induction for muscular dystrophy and other muscle wasting conditions. Philos Trans R Soc B Biol Sci. 2018;373(1738):20160528. 10.1098/rstb.2016.0528 PMC5717528 29203713

[70] Alloush J, Weisleder N (2013). TRIM proteins in therapeutic membrane repair of muscular dystrophy. JAMA Neurol.

[71] Zhao L, Son JS, Wang B, Tian Q, Chen Y, Liu X, et al. Retinoic acid signalling in fibro/adipogenic progenitors robustly enhances muscle regeneration. EBioMedicine. 2020;60:103020. 10.1016/j.ebiom.2020.103020 PMC7519288 32980698

[72] Halade GV, Jin YF, Lindsey ML (2013). Matrix metalloproteinase (MMP)-9: a proximal biomarker for cardiac remodeling and a distal biomarker for inflammation. Pharmacol Ther.

[73] Lourbakos A, Yau N, De Bruijn P, Hiller M, Kozaczynska K, Jean-Baptiste R (2017). Evaluation of serum MMP-9 as predictive biomarker for antisense therapy in Duchenne. Sci Report.

[74] GTEx Portal [Internet]. Available from: https://www.gtexportal.org/home/gene/DMPK. [Cited 2022 Jul 13].

[75] Wei C, Jones K, Timchenko NA, Timchenko L (2013). GSK3β is a new therapeutic target for myotonic dystrophy type 1. Rare Dis.

[76] Wei C, Stock L, Valanejad L, Zalewski ZA, Karns R, Puymirat J (2018). Correction of GSK3β at young age prevents muscle pathology in mice with myotonic dystrophy type 1. FASEB J.

[77] Wang M, Weng W-C, Stock L, Lindquist D, Martinez A, Gourdon G (2019). Correction of glycogen synthase kinase 3β in myotonic dystrophy 1 reduces the mutant RNA and improves postnatal survival of DMSXL mice. Mol Cell Biol.

[78] van Engelen B, Abghari S, Aschrafi A, Bouman S, Cornelissen Y, Glennon J, et al. Cognitive behaviour therapy plus aerobic exercise training to increase activity in patients with myotonic dystrophy type 1 (DM1) compared to usual care (OPTIMISTIC): Study protocol for randomised controlled trial. Trials. 2015;16:224. 10.1186/s13063-015-0737-7 PMC4449962 26002596

