# Supplementary material for: Clinical improvement of DM1 patients reflected by reversal of disease-induced gene expression in blood
Source: BMC Med. 2022 Nov 10;20:395. doi: 10.1186/s12916-022-02591-y (PMC9646470; doi:10.1186/s12916-022-02591-y)
Supplement: Supplementary file 1 — Additional file 1: Table S1. PCR duplicates. [file 12916_2022_2591_MOESM1_ESM.docx]

**Table S1: PCR Duplicates**

| **PCR Sample ID** | **Patient ID** | **Reads in** | **Reads out** | **% Duplicates** |
| --- | --- | --- | --- | --- |
| 3 | D001_V4 | 74255337 | 6717608 | 91 |
| 4 | B053_V2 | 34902678 | 17657186 | 49 |
| 5 | B001_V2 | 32086534 | 19022483 | 41 |
| 6 | D001_V2 | 37216982 | 22233948 | 40 |
| 7 | C048_V4 | 97744825 | 6093127 | 94 |
| 9 | C055_V4 | 26857293 | 4802757 | 82 |
| 10 | B009_V4 | 27127348 | 5821860 | 79 |
| 12 | A005_V2 | 52839750 | 27213328 | 48 |
| 13 | B052_V4 | 46241711 | 9651283 | 79 |
| 14 | B028_V4 | 35160942 | 21099665 | 40 |
| 15 | B001_V4 | 55901222 | 25549654 | 54 |
| 16 | B042_V2 | 30533502 | 16419733 | 46 |
| 17 | B032_V2 | 38685827 | 12594622 | 67 |
| 18 | A017_V2 | 31667720 | 22563298 | 29 |
| 19 | B053_V4 | 34669594 | 22737682 | 34 |
| 20 | B011_V4 | 43683175 | 28303487 | 35 |
| 21 | D003_V4 | 29746212 | 9117004 | 69 |
| 23 | C048_V2 | 40956378 | 14674589 | 64 |
| 24 | C036_V2 | 31094075 | 18619186 | 40 |
| 25 | C039_V2 | 33732128 | 19923385 | 41 |
| 26 | C023_V4 | 87472685 | 8368215 | 90 |
| 27 | C039_V4 | 32324685 | 15233522 | 53 |
| 28 | C036_V4 | 29116832 | 6358863 | 78 |
| 29 | D037_V2 | 50853995 | 9424147 | 81 |
| 30 | D043_V2 | 38565222 | 9848583 | 74 |
| 31 | A023_V2 | 43568775 | 9619931 | 78 |
| 32 | A064_V2 | 52385485 | 9710934 | 81 |
| 33 | B032_V4 | 33748110 | 14557887 | 57 |
| 34 | C014_V4 | 64527362 | 8321368 | 87 |
| 35 | C004_V4 | 45992074 | 21544600 | 53 |
| 36 | C034_V4 | 33772304 | 20752983 | 39 |
| 37 | C014_V2 | 91287051 | 25791002 | 72 |
| 38 | C023_V2 | 32097203 | 9338738 | 71 |
| 39 | C019_V2 | 68218732 | 11297909 | 83 |
| 40 | A005_V4 | 37127933 | 16984404 | 54 |
| 41 | A038_V4 | 32282945 | 16454541 | 49 |
| 42 | A038_V2 | 73595623 | 8512630 | 88 |
| 43 | C004_V2 | 47664760 | 9628878 | 80 |
| 44 | C046_V2 | 32477430 | 16013060 | 51 |
| 45 | C034_V2 | 73271016 | 15781730 | 78 |
| 46 | C055_V2 | 39645604 | 10658203 | 73 |
| 47 | C046_V4 | 70648537 | 7596368 | 89 |
| 48 | D043_V4 | 85026900 | 6356019 | 93 |
| 49 | A064_V4 | 56851191 | 20306373 | 64 |
| 50 | D037_V4 | 33753201 | 23851356 | 29 |
| 51 | A017_V4 | 31416730 | 17668191 | 44 |
| 53 | B052_V2 | 54599613 | 12969826 | 76 |
| 54 | B028_V2 | 75294410 | 7142975 | 91 |
| 55 | A023_V4 | 61933892 | 8854582 | 86 |
| 56 | B042_V4 | 44165589 | 13127682 | 70 |
| 57 | C019_V4 | 42917480 | 9427079 | 78 |
| 58 | B011_V2 | 30187435 | 12024301 | 60 |
| 59 | B009_V2 | 78351372 | 7813368 | 90 |
| 60 | D003_V2 | 42353399 | 18407056 | 57 |

For all the samples included in our analyses, table S1 illustrates the raw PCR counts as well as the percentage of PCR duplicates. Sampling timepoints during the trial can be derived from the Patient ID’s, with respectively V2 and V4 referring to before and after the intervention.
